# Caution with competitive gamification in medical education: unexpected results of a randomised cross-over study

**DOI:** 10.1186/s12909-023-04258-5

**Published:** 2023-04-19

**Authors:** Jacqueline Kirsch, Cord Spreckelsen

**Affiliations:** 1 Medical Clinic, Luisenhospital, Boxgraben 99, 52064 Aachen, Germany; 2 Institute for Medical Statistics, Computer and Data Sciences (IMSID) of the university hospital Jena, Bachstraße 18, Haus 1, 07743 Jena, Germany

**Keywords:** Distance education, Gamification, Evidence-based medicine, e-learning, Competition, Longitudinal learning, Intrinsic motivation

## Abstract

**Background:**

To intrinsically motivate students in the long term, longitudinal e-learning systems combined with repeated testing and competitive gamification seem promising. The effects of this approach have never been closely examined in the field of evidence-based medicine. The authors investigated if a simple, competitive learning application enhances students’ risk competence and intrinsic motivation.

**Methods:**

Participants were 5.-9. semester medical students (*n* = 48), recruited in an elective evidence-based medicine subject and randomly distributed to two groups (group 1: *n* = 23; group 2: *n* = 25). Both accessed a competitive evidence-based medicine quiz game. Following a cross-over design, each group practiced with one of two thematically different questionnaires A or B, before the allocation switched after one month. To analyse whether there was a measurable learning effect in the practiced topics, a paired t-test was performed with quantitative data from 3 e-tests. Students further reported their experience in evaluation surveys.

**Results:**

Students’ improvements in e-test scores after training with the corresponding topics in the learning application can be attributed to chance. Even though the majority enjoyed playing and felt motivated to study, they invested a minimum of time and rejected competition.

**Conclusion:**

The authors found no evidence for benefits of the investigated learning programme on students’ risk competence or on their internal motivation. The majority disapproved the competitive concept, indicating adverse side effects of the applied gamification element. To intrinsically motivate more students, prospective learning programmes should favour complex and collaborative programmes over simple and competitive ones.

## Introduction

Many physicians suffer from statistical illiteracy—their struggle to interpret statistical numbers causes extra expenses in public health systems, harms patients and exposes themselves to manipulation [1]. The “cure” to statistical illiteracy is understanding of evidence-based medicine (EbM) imparted during their studies. Nevertheless, risk assessment and communication are underrepresented in the German medical curriculum [2]. Supporting learning programmes are needed. But what do these programmes require to be easily implemented and spark intrinsic motivation in students? This question has so far not fully been answered in the field of EbM. The answer holds the opportunity to create more effective learning programmes to empower future physicians to treat their patients responsibly.

### Contextual background

To solve clinical problems, medical students need to recall knowledge in the long term. Still, to perform high in tests, they often focus on learning in the short term, hindering the transfer of information to their long-term memory. This is contrasted with intrinsic motivation, which leads students to deal with content independently over a longer period. In medical education, we perceive persistent intrinsic motivation as challenging. Longitudinal learning appears as a valuable approach for stimulating intrinsic motivation and long-term engagement. The pressing problem of statistical illiteracy led us to practically apply a longitudinal learning concept. Physicians experience difficulties interpreting numerical facts [1]. They misjudge diagnostic accuracy and risk or benefit of treatments. For example, they overestimate 5-year survival-rates in cancer-screenings [3], although we can attribute these to lead time bias rather than to mortality reduction [4]. Physicians’ deficiency in risk competence roots in their education—where learning programmes can intervene. A promising attempt constitutes repeated testing. It encourages students to autonomously study and continuously engage with the tested material [5]. Moreover, it increases medical students’ risk competence [2]. With present medical students being continuously confronted with digital media, e-learning is an upcoming topic. A Cochrane review by Vaona et al. shows little evidence of e-learning’s superiority to traditional learning in patient outcome or behaviour of physicians. Yet, it can still be favourable when “reach[ing] a large number of health professionals at a limited cost”(p.18 [6]). This aspect of cost gains importance in longitudinal programmes. According to Festinger’s theory of social comparison (SCT), people aspire to evaluate their own abilities by comparing themselves to others [7]. Responding to this inner urge to compare, a competitive e-learning module appears reasonable. Competitive gamification can successfully increase enjoyment and motivation in medical students and support medical education [8–14]. On the other hand, high competition can correlate with introspection of inferiority or shame, rising stress, anxiety, self-harm and depression [15]. A stressful and strongly competitive learning environment can be linked to suicidal tendencies among medical students [16]. This ambivalent characteristic requires careful handling. In case of a non-mandatory game, positive motivational aspects might prevail. It appears unlikely that a game-based learning app will put much pressure on students. Nevertheless, we need to think critically about whether a competitive game promotes a competitive environment. Affinity to competitive games depends on learning type—some students benefit from competitive learning, while others prefer collaboration. Collaborative learning type medical students outnumber the competitive type [17]. However, the competitive type may be more common in medicine than in other professions, since it is still perceived as a competitive subject [18]. Therefore, we investigated if repeated testing combined with competitive gamification surpasses the effects of repeated testing alone.

### Theoretical background

#### Self-determination theory

“Intrinsic motivation” refers to the established empirical self-determination theory (SDT), developed foremost by Deci and Ryan. It distinguishes extrinsic and intrinsic motivation [19]. Extrinsic motivation is applied through external conditioning. By contrast, intrinsically motivated behaviour itself is rewarding and therefore likely to be maintained without external reinforcements [20]. In this context, motivation aims to satisfy three basic and universal needs: Autonomy, competence, and relatedness [21, 22]. Autonomy describes a behaviour as volitional and coherent with the inner self [23]. Competence enables to effectively master the environment [24]. Relatedness encompasses holding strong and persistent interpersonal connections [25]. The fulfilment of said needs is essential for self-realization, personal growth and mental health [21]. Learning programmes primarily work as extrinsic factors of motivation. One of the first experiments of SDT illustrates the influence of extrinsic rewards on intrinsic motivation [26]: Probands lost genuine interest in a behaviour when they received external monetary rewards. Still, their internal motivation increased when they were verbally appreciated. We assume that the need of competence was satisfied by the positive feedback. Hence, extrinsic motivation can enhance intrinsic motivation when the basic needs are fulfilled. For example, to increase autonomy in students, we should avoid imposing stringent restrictions on them. To satisfy the need of relatedness we should create a safe and caring learning environment where they can connect with others.

#### Social comparison theory

The theory of social comparison (SCT), developed by Festinger [7], alleges an inner desire of people to evaluate their own abilities. In absence of an objective reference point, they fulfil this need by comparing themselves to others [7]. One could therefore interpret that people strive for comparison in the form of competition. However, the readiness to compare decreases as the perceived differences in abilities between individuals increase[7] and depends on whether one feels superior or inferior to the other person. Comparing oneself to those who seem inferior (downward comparison) can enhance subjective well-being [27], especially when oneself is in an unfavourable situation or suffers from depression [28]. Upward comparison involves comparing oneself to someone considered superior [29]. Even though one would assume it therefore evokes feelings of inferiority, it can also lead to self-improvement by following one's role model [30]. These contradictory aspects emphasize that some students could reject a competitive e-learning game, fearing the feeling of inferiority associated with an upward comparison, while others could appreciate it.

#### Gamification

Gamification is frequently applied to improve user motivation in varying fields, for example service [31], management [32], workplace [33, 34], government [35] and – unsurprisingly—also education [36–38] and health care [39]. Gamification can describe anything game-related [40]. For research purpose, it is important to specify. Deterding et al. describe gamification as applying video game elements to non-game systems [41]. Landers emphasized that gamification influences behaviour and attitude, mediating learning outcome [42]. Studies investigating gamification are so various and context-dependent that their results are difficult to generalize. Some find positive effects of gamification, for example increasing engagement [36], creativity and productiveness [43] or motivation [34]. Other studies cannot verify such effects. Hanus and Fox tested a gamified education class, observing less motivation and engagement in the intervention group [44]. Gamification can implement negative emotions in users, who lack the skills to succeed in a task [39]. Leclercq et al. demonstrated the negative impact of losing a competitive challenge on costumer engagement and experience [45]. Interestingly, the loss affected the tested subjects less when they were already integrated into the community. The observed loss aversion might be explained by the unfulfilled need for competence, while the satisfied need for relatedness could compensate in those who were highly engaged in the group. McGraw et al. found loss aversion increasing when a direct comparison was encouraged [46]. Competition, used as a gamification element, shows divergent effects on behaviour and outcome. Hammedi et al. found that implementing a competitive game within costumer service decreased engagement and well-being in employees [33]. De Marcos-Ortega et al. implemented competitive elements in a gamified online class, which increased social interactions and the probability of completing the course [37]. The competitive environment fostered social connection and thus relatedness. Landers et al. found that competition improved creativity and quantity of completed tasks [43]. Callan et al. emphasized that gamification does not necessarily mediate the intended behaviour or lead to the desired outcome [47]. According to Landers, it is insufficient to blindly include random gaming elements, instead we have to carefully consider the psychological effects that selected elements have on behaviour and attitude [32]. We can effectively use competitive gamification when developing an e-learning programme in medical education if we reflect on the psychological impacts of the chosen elements.

#### Related studies

On PubMed, we researched studies with competitive e-learning interventions in medical education. We found reference for different levels of complexity in learning apps. We distinguish simple from complex gamification. Simple gamification includes singular factors of gamification in repeated testing. Convenient features were online quizzes or visualisation of high scores [8, 12]. Van Nuland et al. realized an immediate way of competition in direct online tournaments [13]. As a further video game element, processing time was implemented in grading [12, 13]. In contrast, complex gamification inserts learning content in expanded environments. Respective applications assimilated features of simple gamification, like ranking lists [9, 14] or included operation speed in grading [9]. These items were set into broader context, for example in a practice simulation [14]. Others incorporated multiple-choice-questions (MC-questions) into a team-based, competitive strategy game [10] or a second-life virtual world elimination game [11]. Corell et al. involved students deeper by making them creators of their own quiz challenges [9]. Simple as well as complex gamification affected student’s motivation positively [8–14]. As a secondary positive impact, they enhanced collaboration and communication between students [9, 10, 12, 14]. This contradicts the hypothesis that competitive games create a competitive environment and, consequently, stress in students. Assuming that both levels of complexity can successfully be used, we favour the simple programmes as they require less investment in time and cost. Even though competitive e-learning programmes cover diverse subjects of the medical curriculum, they have so far failed to address EbM.

#### Developing the intervention

**Fig. 1:**
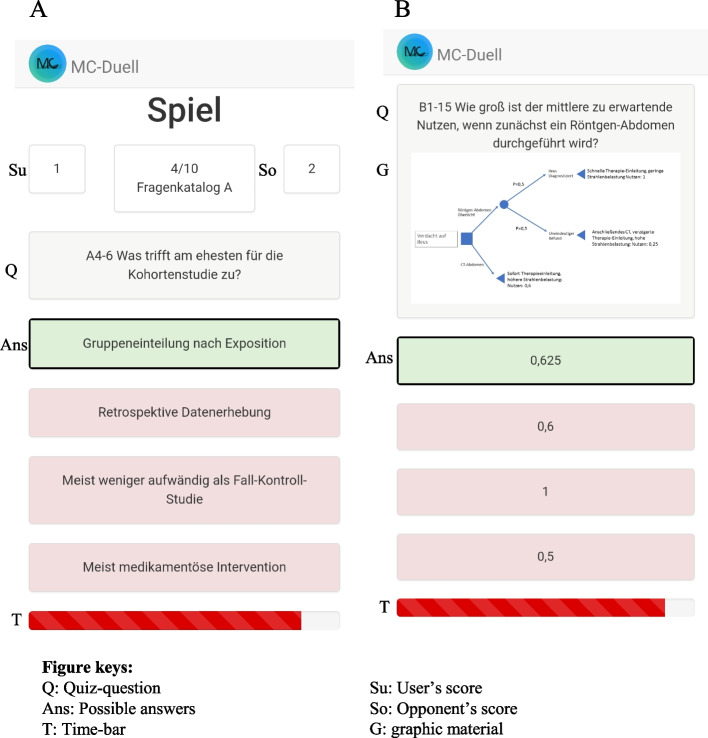
Mobile view on user’s-screen in MC-Duell. Legend: **A** shows a correctly answered question from questionnaire A. **B** shows a correcly answered question with graphic material from questionnaire B. The correct answer is highlighted in green. During the match, both of the players’ scores and the time-bar are visualized

We designed a simple EbM e-learning tool to enhance medical students’ intrinsic motivation. As a longitudinal learning programme, we developed the competitive online quiz MC-Duell. In this programme, students compete directly answering EbM-themed single-choice-questions in real-time matches (s. Figure 1 A-B). As a primary outcome, we investigate MC-Duell’s influence on students’ test performance. As a secondary outcome, we analyse its effects on their motivation to study EbM.

## Methods

### The intervention

The Department of medical Informatics of RWTH Aachen University developed MC-Duell, a competitive online quiz, as an AngularJS web application, deployed on an ExpressJS webserver. Prior to starting a quiz match, participants freely pick how many rounds to play, each consisting of one single-choice-question with four answer options, as seen in Fig. 1. Students decide on whether items with graphical material are included (Fig. 1B). To start a duel, participants need to find an opponent. For joining someone in their match, students could either use the displayed internet link or scan a QR-code. Via a Websocket-protocol, players compete in real time. Each single-choice-question is answered within a time limit of two minutes. Figure 1 shows how, once a user completes a question, the correct answer is highlighted in green. After finishing all quiz items, final scores are displayed. The player who answered the most questions correctly or, in case of a tar, the fastest player wins. We realized different features of gamification in the app. Some of them account for its competitive character: Direct competition in real-time matches and game scores visualised during and after the match. A running out time-bar is displayed with each question (Fig. 1). The concise phrasing, resembling commercial quiz games, makes the rounds quickly playable. After each round, the correct answer is revealed immediately as a close-meshed reward-system. Video games frequently implement this technique of classical conditioning.

### Quiz items

We created over 550 single-choice-questions. Figure 1A shows a sample question, Fig. 1B a question with graphical material. The items’ content was based on the third semesters’ EbM course of the medical curriculum of RWTH Aachen University. Within quality control, a feedback panel of EbM-experienced members of the Department of medical Informatics reviewed the items. For the cross-over design, we assigned each question to one of two questionnaires. Table 1 shows how we divided the questions into 9 different topics, which we randomly allocated to two questionnaires A and B. We randomly extracted 30 questions from each questionnaire and inserted them in one of 3 e-tests. For the primary outcome, we analysed participants’ e-test scores.

**Table 1:** MC-Duell’s questionnaires

A		B	
Randomisation, stratification and blinding	13	Decision analysis	20
Evidence-based medicine	1	Researching clinical problems in scientific literature	30
M-health, hospital information system, guidelines, CDSS and data privacy	42	Evaluating quality of scientific literature	17
Study design	49	Bias	52
Extracting relevant data	5		
Items with graphic material	10	Items with graphic material	14
Total	110	Total	119

Final MC-Duell questionnaires A and B with their topics and respective number of questions. In summary, the total number of questions and the number of questions with graphic material are shown

### The final questionnaires

Table 1 illustrates the distribution of quiz items into two questionnaires A and B. Each consisted of 5 (A) or 4 (B) topics and comprised 110 (A) or 119 (B) questions. It is apparent that the final questionnaires are smaller than the original pool of questions. This is due to the circumstance that we originally concepted four questionnaires (s. Discussion: Adjustment in study design). In January 2019, the final questionnaires were converted into a Microsoft Excel sheet, except for the graphic items which were converted into an XML-file. Both were inserted into MC-Duell. We created two access links for the app, each for one of the two questionnaires. When using these accesses, the questions of the corresponding questionnaire were presented as quiz items in MC-Duell.

### Recruiting participants

We recruited participants in the elective subject “Curing statistical Illiteracy” for 5.-9. semester medical students of RWTH Aachen University. This course pursues a multimodal, longitudinal and spaced e-learning concept to improve students’ risk competence. Apart from MC-Duell, the subject contains learning videos, scripts and e-tests. It should be noted, that EbM was part of third semesters’ curriculum, so participants were already experienced. We tested MC-Duell twice in the elective subject. The first time, in the summer semester 2019, 36 students (10 males and 26 females) signed up. In the second term, in winter semester 2019/2020, 12 females participated. In total, *n* = 48 students participated. We stratified them according to the semester they attended and afterwards randomly divided them into two groups 1 and 2. In summer, there were 17 students in group 1 and 19 in group 2. In winter, there were 6 students in each group.

### Cross-over study design

**Fig. 2:**
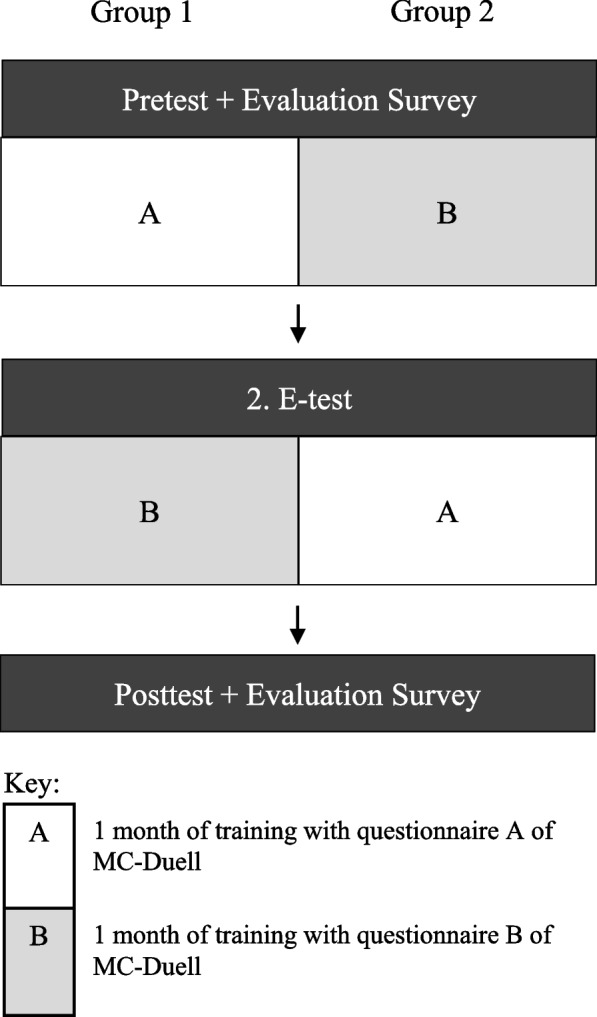
Illustration of the cross-over study design. Legend: The figure shows the timeline of the study with e-tests, surveys and training phases for both groups 1 and 2

Figure 2 illustrates the cross-over design, in which both groups consecutively trained with the two different questionnaires of MC-Duell. After a pretest, each group accessed their allocated questionnaire. Group 1 trained with questionnaire A and group 2 with questionnaire B. After one month, they took the second e-test. Subsequently, the allocation of group and questionnaire switched. During the second month, group 1 trained with questionnaire B and group 2 with questionnaire A. Conclusively, participants took the posttest.

### E-tests and evaluation surveys

Every e-test consisted of 20 items with 10 items extracted from each questionnaire, A and B. Students could accomplish them in 10–15 min. They accessed the e-tests sequentially for one month, creating a spaced learning system. To qualify for the next e-test, students had to play at least one round of their previous MC-Duell questionnaire. For the secondary outcome, students filled in an evaluation survey simultaneously to pretest and posttest. It contained 18 items which they rated on a 4-point Likert scale, depending on how much they agreed to a given statement (1 = strongly disagree, 4 = strongly agree). The survey addressed the impact of EbM in their curriculum and later profession. It included self-assessment of their long-term retention in EbM and MC-Duell’s influence on it, how they perceived competition and the app’s effect on their motivation. In addition, students answered 3 free text questions. In the pretest, they described what they expected of MC-Duell. In the posttest, they drafted what they liked and what they thought could be improved. They further recorded their time investment per week, varying between “less than 1 h” to “over 10 h”. Students accessed e-tests, evaluation surveys and URLs to MC-Duell on the learning management system (LMS) Moodle.

### Methods of analysis

We analysed data using SPSS software (version 24).

#### E-test: descriptive statistics

We described the two groups’ total test scores (out of 20 achievable points), along with test performances in each of the two questionnaires’ subsections (part A and B, each 10 achievable points), regarding mean, standard deviation (SD) and 95% confidence interval (CI).

#### E-test: statistic inference

We performed a paired t-test comparing the first e-test of each group to its second and third one. We considered total test score and subsections A and B. To adjust the significance level, we used the Bonferroni correction, α_adj =_ 0.004. We limited the analysis to completely answered first attempts of participants. Further tries were not included, otherwise previous processing of the same item could distort the results.

#### Evaluation survey

We described the results of the evaluation survey using absolute and relative numbers. We compared pretest and posttest surveys using an ANOVA. We adjusted the significance level with the Bonferroni correction, α_adj_ = 0.003.

#### Dropouts

We included dropouts, resembling an intention to treat analysis, by incorporating their e-test scores in the descriptive and inferential statistics. Yet, the t-test does not represent students who completed less that 2 e-tests. Participants withdrew from the trail when they ceased training with MC-Duell and/or taking e-tests. If students failed to adhere to the schedule, we encouraged them to contact the supervision. After receiving access to the missed material, they continued with the programme.

### Data safety

In the beginning of the subject, we informed students about the use of collected information for study purpose. We recorded the results on the LMS. Before further analysis of e-tests, we pseudonymized the participants using randomized numbers. The LMS automatically anonymized the results of the evaluation survey.

## Results

### E-tests

#### Descriptive statistics

The LMS recorded 49 attempts of taking the pretest. Three students of each group took it twice. One student from group 1 answered inchoate. We included only the 42 fully completed first attempts, consisting of 21 students from each group. As shown in the first column of Table 2, students in group 1 scored a mean of 11.86 points (95% CI: 10.61–13.11), members of group 2 a mean of 11.81 (10.88–12.74). In part A, group 1 achieved 5.48 points (4.76–6.19), group 2 surpassed them with 6.33 (5.65–7.01). In part B, group 1 exceeded with 6.38 (5.55–7.21), group 2 accomplished a mean of 5.48 (4.91–6.05). After one month, the number of participants halved itself: In a total 23 students, 12 from group 1 and 11 from group 2, took the second e-test. One student from each group answered inchoate, making 21 adequate attempts. As you can see in the second column of Table 2, both groups’ total test performances decreased. Group 1 scored 10.91 (8.86–12.96), meanwhile group 2 obtained 10.70 points (8.29–13.11). In part A, group 1 reached 5.18 points (4.03–6.34). The second groups’ performance dropped to 5.00 (3.25–6.75). In part B, group 1 scored 5.73 points (4.45–7.01), whilst group 2 increased to 5.70 points (4.05–7.35). After the second month of practice, 24 participants attempted the posttest, 14 students from group 1 and 10 from group 2. One student from group 2 and 2 students from group 1 attended the posttest without attempting e-test 2, one of the last did also not answer the posttest appropriately. Therefore, 23 students completed the posttest adequately, 13 of them attending group 1 and 10 group 2. Turning now to column three of Table 2, both groups increased their total test performances. Group 1 achieved a mean of 13.77 (12.04–15.50), group two reached 12.50 (10.81–14.19). Group 1 increased in part A to 7.08 (5.91–8.25) and group 2 scored 6.30 (5.08–7.52). Analysing part B, group 1 attained 6.69 (5.82–7.56) and group 2 6.20 points (5.09–7.31).

**Table 2:** Descriptive analysis of students’ e-test results

	E-test 1				E-test 2				E-test 3			
	Mean	SD	95% CI		Mean	SD	95% CI		Mean	SD	95% CI	
			Lower limit	Upper limit			Lower limit	Upper limit			Lower limit	Upper limit
Group 1												
Total	11.86	2.74	10.61	13.11	10.91	3.05	8.86	12.96	13.77	2.86	12.04	15.50
A	5.48	1.57	4.76	6.19	5.18	1.72	4.03	6.34	7.08	1.93	5.91	8.25
B	6.38	1.83	5.55	7.21	5.73	1.90	4.45	7.01	6.69	1.44	5.82	7.56
Group 2												
Total	11.81	2.04	10.88	12.74	10.70	3.37	8.29	13.11	12.50	2.37	10.81	14.19
A	6.33	1.49	5.65	7.01	5.00	2.45	3.25	6.75	6.30	1.70	5.08	7.52
B	5.48	1.25	4.91	6.05	5.70	2.31	4.05	7.35	6.20	1.55	5.09	7.31

Students’ e-test results regarding total test scores (out of 20 achievable points) and scores in subsections A and B (out of 10 achievable points). We described mean, standard deviation (SD) and 95% confidence interval (CI). We only included completely answered first attempts. Number of students included in each e-test: e-test 1: 42; e-test-2: 21; e-test 3: 23

#### Statistic inference: paired t-test

**Fig. 3:**
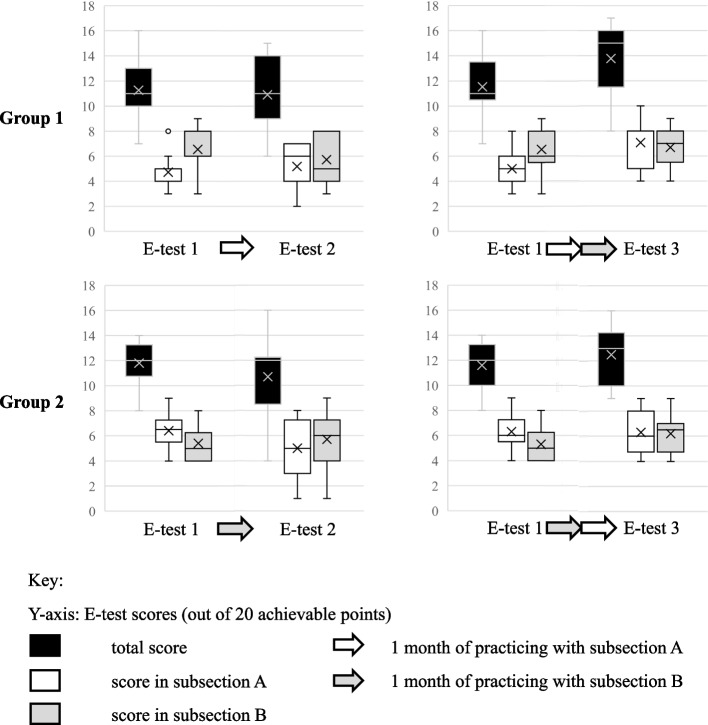
Box-plot illustration of the e-test scores. Legend: Data of students’ e-test results regarding scores of the subsections A and B plus total. Data from all 24 students who completed more than 1 e-test is analysed in the paired t-test. In total, 20 points were archievable. In subsections A and B, 10 points were achievable

We compared the results of pretest and e-test 2. As the left box-plots in Fig. 3 illustrate, neither of the changes in test scores in the two groups were significant (α_adj_ = 0.004). Group 1 decreased their performance in total from a mean of 11.27 to 10.91 points (*P* = 0.57). In part B, they dropped from 6.55 to 5.73 (*P* = 0.13), increasing exclusively in part A from 4.73 to 5.18 (*P* = 0.41). Concerning group 2, the test scores in total declined from 11.80 to 10.70 (*P* = 0.39), together with the performance in part A, in which they deteriorated from 6.40 to 5.00 (*P* = 0.17). They improved only in part B from 5.40 in the pretest to 5.70 points in e-test 2 (*P* = 0.71). Let us now turn to the right box-plots in Fig. 3 to compare pretest with posttest. Group 1 scored better in total from 11.54 to 13.77 (*P* = 0.08) as well as in part B in which they ameliorated from 6.54 in the pretest to 6.69 (*P* = 0.83). In part A they enhanced from 5.00 points to 7.08 (*P* = 0,01). Group 2 increased in total from 11.60 to 12.50 (*P* = 0.13). Improving in subsection B from 5.30 in the pretest to 6.20 points in the posttest (*P* = 0.11), they maintained a score of 6.30 in subsection A. 8 students from group 1 and 10 from group 2 were not represented in the t-test, because they submitted only 1 e-test. The same applies for 3 from each group who did not complete any test. In contrast, the statistic included 11 participants who missed the deadlines and continued the course after contacting the supervision. In summary, after one month of practice, both groups increased in the subsection they trained with, whilst decreasing in the other and in total score. From pretest to posttest, the groups enhanced their performance in total and in both subsections (except for group 2 maintaining their results in part A). These differences, however, can be explained by chance.

### Evaluation survey

**Fig. 4:**
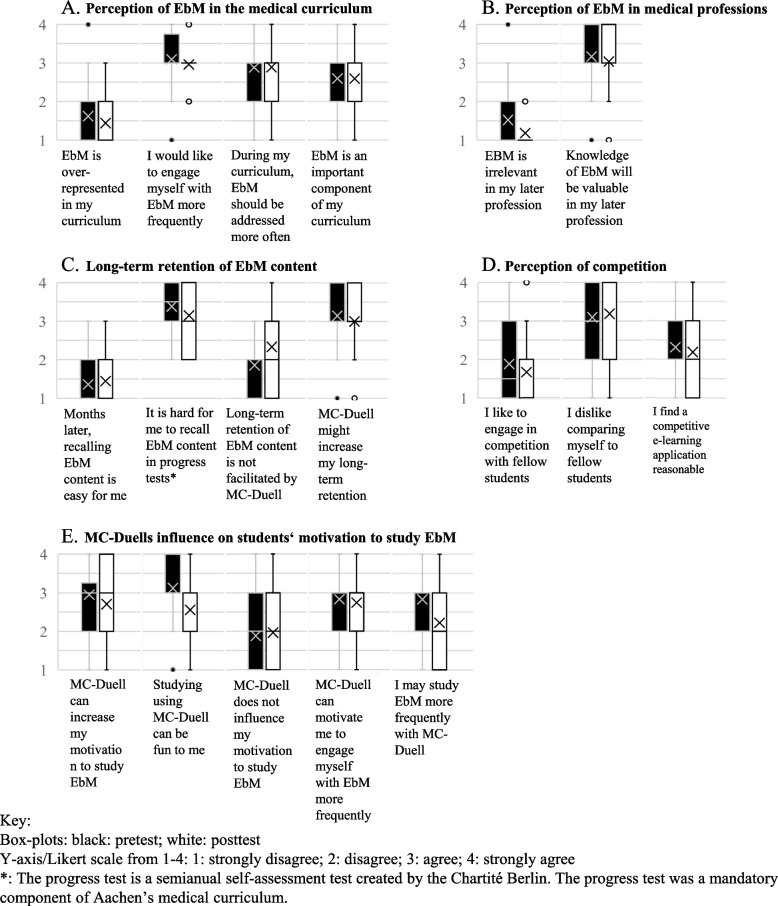
Box-plot illustration of the evaluation survey responses. Legend: Data of the 2 evaluation surveys, including 42 responding students in the pretest, 27 respondents to the posttest who evaluated their experiences on a Likert scale from 1–4

Forty-two participants took the first evaluation survey (31 of the first term and 11 of the second). Twenty-seven students completed the final survey (19 from the first term and 8 from the second). According to the ANOVA, we can attribute the changes between pretest and posttest to chance (α_adj_ = 0.003). Unless specified otherwise, we describe the results of the pretest survey more detailed below, due to higher response rate. For further information about both surveys, see Fig. 4.

#### Likert scale items and time expenditure

Regarding the weekly time expenditure, the larger part of 25 students (92.6%) played MC-Duell less than 1 h, the remaining 2 (7.4%) 1-2 h. The time spent was minor with over nine-tenths engaging themselves less than 1 h a week. The majority of 36 students (85.8%) did not find EbM overrepresented in their curriculum. Twenty-nine participants (69.0%) thought, EbM should be addressed more often. Thirty-seven (88.1%) wanted to engage themselves with EbM more frequently. There were divided views on whether EbM was an important component of their curriculum: 23 (54.8%) consented whilst 19 (45.2%) dissented. A great part aspired to engage themselves more extensively with EbM. They perceived it as essential in the medical curriculum, as illustrated in Fig. 4A, and found it important for their later carriers (see Fig. 4B). Thirty-five students (83.3%) valued EbM knowledge in their later profession, 6 students (14.2%) found EbM irrelevant in their future occupation. Despite this appreciation, most rated their risk competence as insufficient, illustrated in Fig. 4C. A minority of 3 students (7.1%) considered recalling EbM content months later as easy, opposing 39 students (92.8%) who contradicted. After the intervention, this fractions hardly changed. Twenty-six students (96.0%) still had problems recalling the content (*P* = 0.56). Thirty-seven participants (88.1%) found it hard to remember EbM content in progress tests (a semi-annual self-assessment test for medical students by Charité Berlin). Furthermore, we investigated if students thought, MC-Duell could increase their long-term retention of EbM content. This item appeared twice, once in negation. We combined the ratings for both items for analysis: In the pretest, an average of 36 (85.8%) agreed or strongly agreed. In the posttest, this proportion shrank to 17.5 (64.8%) (*P* = 0.03). Figure 4D shows students’ perception of competition. The mass of 31 participants (73.8%) did not enjoy engaging themselves in competition with fellow students. Thirty-one students (73.8%) disliked comparing themselves to their classmates. Students had dissenting opinions about a competitive e-learning application: 17 (40.5%) found it reasonable whilst a slight majority of 25 students (59.5%) contradicted. In contrast to this aversion to competition, students rated MC-Duell’s influence on their motivation as more beneficial. This can also be seen in Fig. 4E. In the pretest, 31 students (73.8%) thought that MC-Duell could increase their motivation to study EbM. In the posttest, this number shrank to 15 students (55.5%) (*P* = 0.26). The number of participants who thought, MC-Duell would not influence it remained relatively stable from 11 participants (26.1%) in the pretest to 8 (29.6%) in the posttest (*P* = 0.73). Thirty participants (71.4%) supposed MC-Duell could activate them to study EbM more frequently. After the training, only 10 students (37.0%) agreed (*P* = 0.01). The relative number of students who thought using MC-Duell might motivate them to engage themselves with EbM more often stayed almost constant: 28 participants (66.6%) in the pretest and 17 (62.9%) in the posttest (*P* = 0.66). Thirty-six students (85.7%) assumed they would enjoy using MC-Duell. The percentage of students who claimed to have enjoyed it was lower in the posttest with 15 (55,5%) (*P* = 0,01). In both surveys, over half of the participants assumed MC-Duell improved their long-term retention of EbM content. The majority disapproved competition in medical education. Yet, over half of the surveyed enjoyed MC-Duell and found it motivated them to study EbM.

#### Free text questions

In the pretest, 17 students formulated what they anticipated from MC-Duell. Most frequently expected with 7 references were increasing long-term retention, as well as consolidating and revising EbM content in a playful approach. Three students hoped to increase their motivation. Once mentioned were learning new EbM content or advance in recalling it, recognizing knowledge gaps, exchanging with others, implementing acquired statistic skills to writing a scientific thesis, learning from mistakes and increase self-confidence in risk competence. In the posttest, 6 respondents drafted what they enjoyed about MC-Duell. Leading answer with 3 mentions was its playful approach on EbM. Each two times appreciated were the fun in playing it and its potential use for practicing EbM before tests. Once acknowledged were the freely pickable number of rounds, the time limit, its interactivity along with its playability on the side and on the move. Simultaneously, 12 students described what they disliked or could be improved. The majority of 9 participants found it inconvenient, that a partner is indispensable to play MC-Duell. Instead, they suggested adding a single-player mode, beating their own best performance, buffering the game score until an opponent joins or playing against a computer. Four students criticised MC-Duell’s exclusive availability via the intranet of the university. The time limit was considered problematic twice because it kept students from rethinking the answers properly. Two participants asked for explanations for right and wrong answers. One time it was faulted that the same questions appeared twice in one match and that the items were unoriginal. Once animadverted was the competitive character. Furthermore, one participant asked for a scripted synopsis.

## Discussion

### Potential sources of bias

#### Comparability of e-tests and questionnaires

We allocated different quiz items to topics and topics later to questionnaires. To create the e-tests, we extracted questions from the questionnaires. Even though allocation and extraction happened randomized, we cannot fully eliminate bias. It remains debatable whether questionnaires or e-tests are fully comparable.

#### Technical issue

Surprisingly, 3 participants finished the posttest without attempting e-test 2. A technical error of the LMS may explain this circumvention of programmed conditions. Presumably, students could start an e-test without editing the items and still manually marking it as finished.

#### Selection bias

Since we recruited the participants in an elective EbM subject, selection bias could be involved. We may neglect this factor in the quantitative statistics, but it could distort the qualitative analysis of the free text survey questions.

### Adjustment in study design

We originally created two more questionnaires of MC-Duell. In our initial study design, we included two more e-tests and training units (e-test 1 contained 10 further questions that, however, were not analysed since they addressed topics from an additional questionnaire). Our ambition was to investigate the app enclosed in a multimodal e-learning programme. Due to time-dependent limitations, the testing was foreshortened to only two months. Therefore, we abandoned the further test phases, as the training periods would otherwise have been too short. Students received access to the additional material after completing the posttest. In future investigations, we hope to test MC-Duell in an expanded learning programme.

### Validity of self-assessment

Students’ ability of self-assessment of their performances is often underestimated. Yet, self-appraisal-based evaluation tools in medical education produce valid assessment [48]. We can therefore use them to evaluate medical curricula [49]. Applying this consideration to the results of the survey, we may assume positive effects of MC-Duell on students’ long-term memory that are not verifiable in the e-tests. A limitation of this assumption is that over 90% still reported difficulties in recalling EbM content after the intervention.

### Explanation for dropouts

One source of weakness in this study is the relatively large number of dropouts with small sampling size. The rigid timetable may have contributed to participants dropping out. Presumptively, a larger number of students fell behind the schedule than those who contacted the supervision. It is conceivable that the withdrawal from the study and the minimal time commitment share a common cause. We hypothesise that MC-Duell did not intrinsically motivate students in the long term to engage with the learning programme.

### Suggestions for improvement—how to make it work

Over 90% of the surveyed used MC-Duell “less than 1 h” a week. On the one hand, the gamification programme aspires to be playable with minor time effort. On the other hand, “less than 1 h” was the minimal option, making a floor effect possible. Completing at least one round was the only condition to advancing to the next e-test (thereby passing the course). It is conceivable that some of those represented in this category trained only one round with each questionnaire. If higher time investment could have led to major effects in the t-test remains unclear. Yet, since the programme aims to increase students’ intrinsic motivation to engage with the content more often, it may have failed in its purpose. We identified 3 main aspects of MC-Duell which potentially increase engagement and time commitment in students.

#### Respond to the distribution of learning types and the need for relatedness

Hammedi et al. reported negative effects of competition on frontline employees’ engagement and well-being [33]. Attitudes expressed by the employees in their interviews and those of the students in our evaluation survey appear similar. In both cases, a mediating effect of their negative attitude towards competition on the outcome can be discussed. This rejection of competition corresponds with the predominance of collaborative over competitive learning type [17]. Presumably, we can address a greater proportion if we abandon the competitive approach in favour of cooperation. Applying this to MC-Duell, students could form teams to solve quiz items together by exchanging in-game messages. Still, satisfying the need of relatedness must not necessarily mean abandoning competition. Several studies showed positive effects of competition on communication and cooperation: In the general practice simulation game investigated by Hannig et al., communication about gaming strategies led to constructive cooperation between participants [14]. Contrary to MC-Duell, their programme abandoned MC-questions. They incorporated leader boards as competitive element. In their evaluation survey, self-estimated knowledge improved significantly. An immunology game tested by Corell et al. also enhanced collaboration and conversation [9]. They observed a significant gain in test performance, satisfaction and motivation. They also included ranking lists and implemented processing time in grading. Moreover, their app offered versatile opportunities for student participation, allowing them to create challenges of their own. Felszeghy et al. provided histology online quizzes on a web-based gamification platform [12]. They could not show significant effects in the outcome-based tests compared to the previous year without the intervention. Still, the participants approved the competitive concept. Students preferred the team-based mode over the individual mode. But combining competition and collaboration can also have negative effects on engagement, as shown by Leclercq et al. [45]. A possible explanation might be a role conflict between competitor and co-operator. The unfulfilled need for relatedness in students may be one reason why students did not develop intrinsic motivation. Another explanation could be that direct real-time matches caused loss aversion. A study by Worm and Buch supports this idea [8]. In their competitive biology quiz, students competed indirectly, comparing their own score to their peers’ and to high score, thus avoiding a direct win/lose decision. Compared to a non-competitive quiz, they observed a significant enhancement in test performance. Loss aversion could have particular relevance in medicine, where students are under high pressure to perform. When developing future learning programmes in medical education, we can respond to the need for relatedness and the distribution of learning types by applying cooperative e-learning systems. Competition too can fulfil relatedness and enhance intrinsic motivation and collaboration, when handled with care.

#### Higher usability

From the survey, we identify two issues as particularly inconvenient: Autonomously finding a partner and the limited access to MC-Duell via the university's intranet. We also assume that the rigid schedule contributed to dropouts. These circumstances lower the flexibility in MC-Duell and diminish enjoyment. It is possible that these external restrictions left students’ need for autonomy unfulfilled and thus decreased the intrinsic motivation. We investigated how other studies increased usability. Worm and Buch renounced real-time matches [8]. Hannig et al. operated without direct competition, using leader boards instead [14]. Resembling the MC-Duell study, van Nuland et al. imparted anatomical content in an e-learning app, mainly containing MC-questions [13]. In a cross-over design, they detected a positive effect in test performance in the competitive groups. They scheduled online tournaments, thereby simplifying the search for an opponent. Nevertheless, it diminished the flexibility and autonomy of students. In summary, finding an opponent can be circumvented by abandoning direct matches or by scheduling appointments. An alternative option, as proposed in the survey, is adding a single player mode. Turning now to the issue of accessing the learning programme, transferring MC-Duell to a different server would extend its availability beyond the intranet.

#### Increase complexity

It is noticeable that most studies investigating competitive e-learning interventions in medical education produce an outcome favouring competitive programmes. Publication bias may explain the lack of studies with negative outcome. Simple gamification could be particularly affected: We found fewer studies in this category, even though their lower development effort may suggest that more were originally conducted. It seems possible that complex applications are better at motivating students. The findings of our evaluation survey also indicate students’ wish for increased complexity. Some of the surveyed suggested adding in-game explanations. In literature, complex gamification shows positive influence on students’ engagement and collaborative exchange: Janssen et al. inserted anatomy-dependent MC-questions into a team-based competitive strategy game [10]. Students perceived the game as challenging, engaging and enjoyable, appreciating especially the competitive approach. Lorenzo-Alvarez et al. inserted MC-questions into a second-life virtual world in which students competed in an elimination game [11]. Compared to a control group, they scored significantly higher in a post-exposure test and rated the second life as a positive experience. We suppose that our version of simple gamification was not sufficient to intrinsically motivate students in the long term. Presumptively, a complex learning application would have been worth its extra expenditure. Enhancing MC-Duell’s complexity, we could follow Corell et al. [9] and increase student participation. To involve students deeper, we could have them create their own MC-items to gain points and generate or visualize over-all gaming scores.

## Conclusion

This study investigated a competitive e-learning quiz application to “cure” medical students’ statistical illiteracy, aiming to increase students’ intrinsic motivation to engage with EbM in the long term. We found no evidence of the tested simple gamification programme increasing medical students’ risk competence. Changes in test scores can be attributed to chance. There was no indication that it improved their intrinsic motivation. Even though students valued EbM as a major skill in curriculum and profession, they perceived their own risk competence as poor. On the one hand, more than half of the participants enjoyed using MC-Duell and assumed, it would improve their risk competence. On the other hand, they especially rejected the competitive approach and showed low time investment. Nevertheless, we learned a valuable lesson for upcoming studies. When developing prospective longitudinal learning interventions in medical education, the use of competitive gamification must be carefully evaluated. Instead, a collaborative approach may be contemplated. To increase time investment and lower dropouts, it appears promising to increase usability, for example by avoiding real-time matches. In addition, it can be beneficial to increase the complexity of the applications. By incorporating these principles, we hope to design effective learning applications in the future that intrinsically motivate students to become risk competent health professionals.

## Data Availability

The datasets used and analysed during the current study are available from the corresponding author on reasonable request.

## Abbreviations

App: Application
CDSS: Clinical decision support system
CI: Confidence interval
EbM: Evidence-based medicine
E-learning: Electronic learning
Et al.: Et alia
E-test: Electronic test
Fig.: Figure
LMS: Learning management system
MC: Multiple-choice
p.: Page
s.: See
SCT: Social comparison theory
SD: Standard deviation
SDT: Self-determination theory
